# Supplementary material for: DNA Methylation Is a Potential Biomarker for Cardiometabolic Health in Mexican Children and Adolescents
Source: Epigenomes. 2023 Feb 3;7(1):4. doi: 10.3390/epigenomes7010004 (PMC9944859; doi:10.3390/epigenomes7010004)
Supplement: Supplementary file 1 [file epigenomes-07-00004-s001.zip › epigenomes-2190267-supplementary.pdf]

Supplementary Table S1: Spearman's rank correlation coefficients between DNAm z-score at LINE-1 CpG sites:

	LINE-1 site 1	LINE-1 site 2	LINE-1 site 3	LINE-1 site 4
LINE-1 site 1		0.69 (<0.0001)	0.57 (<0.0001)	0.31 (<0.0001)
LINE-1 site 2	0.69 (<0.0001)		0.79 (<0.0001)	0.55 (<0.0001)
LINE-1 site 3	0.57(<0.0001)	0.79 (<0.0001)		0.59 (<0.0001)

Values presented are Spearman's rank correlation coefficients (*p*)

Supplementary Table S2: Spearman's rank correlation coefficients between DNAm z-score at *11β-HSD-2* CpG sites*:*

	11β-HSD-2 site 1	*11β-HSD-2* site 2	*11β-HSD-2* site 3	*11β-HSD-2* site 4	*11β-HSD-2* site 5
11β-HSD-2 site 1		0.29 (<0.0001)	0.69 (<0.0001)	-0.37 (<0.0001)	0.16 (0.0131)
11β-HSD-2 site 2	0.29 (<0.0001)		0.41 (<0.0001)	-0.08 (0.2556)	-0.03 (0.6497)
11β-HSD-2 site 3	0.69 (<0.0001)	0.41 (<0.0001)		-0.27 (<0.0001)	0.10 (0.1265)
11β-HSD-2 site 4	-0.37 (<0.0001)	-0.08 (0.2556)	-0.27 (<0.0001)		-0.20 (0.0022)

Values presented are Spearman's rank correlation coefficients (*p*)

Supplementary Table S3: Spearman's rank correlation coefficients between DNAm z-score at *H19* CpG sites:

	H19 site 1	*H19* site 2	*H19* site 3	*H19* site 4
H19 site 1		0.26 (<0.0001)	0.44 (<0.0001)	0.94 (<0.0001)
H19 site 2	0.26 (<0.0001)		0.84 (<0.0001)	0.17 (<0.0001)
H19 site 3	0.44 (<0.0001)	0.84 (<0.0001)		0.37 (<0.0001)

Values presented are Spearman's rank correlation coefficients (*p*)

Supplementary Table S4: Spearman's rank correlation coefficients between DNAm z-score at *PPAR-α* CpG sites*:*

	PPAR-α site 2
PPAR-α site 1	0.47 (<0.0001)

Values presented are Spearman's rank correlation coefficients (*p*)

Supplementary Table S5: Associations between DNAm z-score at LINE- 1 and Repeated Measures of Cardiometabolic Risk Factors using Mixed-effects Models Adjusting for Pubertal Onset (*n*=242):

	LINE-1 z-score at site 1		LINE-1 score at site 2		LINE-1 score at site 3		LINE-1 score at site 4	
	**Estimate (SE)**	**P-value**	**Estimate (SE)**	**P-value**	**Estimate (SE)**	**P-value**	**Estimate (SE)**	**P-value**
**Waist circumference (cm)** (Total number of observations=441; of which 43 (17.77%) subjects had one measurement, and 199 (82.23%) subjects had two measurements)								
**Model 1**	-0.5960 (1.0435)	0.5684	1.1418 (1.4217)	0.4227	-0.4783 (1.1510)	0.6781	0.2997 (0.9013)	0.7398
**Model 2**	0.6325 (1.0098)	0.5317	1.0269 (1.3678)	0.4535	-1.9266 (1.1252)	0.0881	0.3154 (0.8699)	0.7172
**Systolic blood pressure (mmHg)** (Total number of observations=441; of which 43 (17.77%) subjects had one measurement, and 199 (82.23%) subjects had two measurements)								
**Model 1**	-0.4560 (0.8541)	0.5939	-0.1855 (1.1698)	0.8741	0.1632 (0.9435)	0.8628	0.9703 (0.7361)	0.1887
**Model 2**	-0.6264 (0.8602)	0.4672	0.2907 (1.1720)	0.8043	-0.3020 (0.9613)	0.7537	0.8606 (0.7375)	0.2444
**Diastolic blood pressure (mmHg)** (Total number of observations=441; of which 43 (17.77%) subjects had one measurement, and 199 (82.23%) subjects had two measurements)								
**Model 1**	-0.5185 (0.5769)	0.3697	0.1316 (0.7927)	0.8682	0.2271 (0.6379)	0.7221	0.3619 (0.4966)	0.4669
**Model 2**	-0.4927 (0.5769)	0.3940	0.1524 (0.7887)	0.8469	-0.09831 (0.6458)	0.8791	0.3551 (0.4932)	0.4723
**Log transformed fasting glucose (mg/dL)** (Total number of observations=438; of which 46 (19.01%) subjects had one measurement, and 196 (80.99%) subjects had two measurements)								
**Model 1**	-0.01570 (0.007838)	0.0463	0.02427 (0.01086)	0.0263	-0.00357 (0.008708)	0.6825	-0.00361 (0.006726)	0.5917
**Model 2**	-0.02747 (0.008217)	0.0010*	0.02862 (0.01124)	0.0115	0.008495 (0.009213)	0.3575	-0.00145 (0.007023)	0.8362
**Log transformed high-density lipoprotein cholesterol (mg/dL)** (Total number of observations=438; of which 46 (19.01%) subjects had one measurement, and 196 (80.99%) subjects had two measurements)								
**Model 1**	0.02078 (0.01893)	0.2733	-0.02664 (0.02610)	0.3083	0.01023 (0.02099)	0.6265	-0.01677 (0.01627)	0.3039
**Model 2**	-0.00710 (0.02059)	0.7305	-0.02295 (0.02796)	0.4127	0.04707 (0.02299)	0.0417	-0.00636 (0.01771)	0.7197
**Log transformed triglycerides (mg/dL)** (Total number of observations=438; of which 46 (19.01%) subjects had one measurement, and 196 (80.99%) subjects had two measurements)								
**Model 1**	-0.05170 (0.04055)	0.2035	-0.03424 (0.05541)	0.5372	0.05445 (0.04481)	0.2255	-0.00392 (0.03498)	0.9109
**Model 2**	-0.02828 (0.03955)	0.4752	-0.04454 (0.05388)	0.4093	0.05366 (0.04424)	0.2263	0.009718 (0.03392)	0.7747

- Long interspersed nuclear elements (LINE-1)
- Model 1 includes LINE-1 z-score at CpG site 1, 2, 3, and 4 as fixed effects, and compound symmetry matrix structure to model the covariance structure of the repeated measurements for each outcome
- Model 2 is additionally adjusted for the following fixed effects: age, sex, duration of breastfeeding, and pubertal onset

* $p < 0.008$

Supplementary Table S6: Associations between DNAm z-score at *11β-HSD-2* and Repeated Measures of Cardiometabolic Risk Factors using Mixed-effects Models Adjusting for Pubertal Onset (*n*=229):

	11β-HSD-2 **score at site 1**		***11β-HSD-2*** **score at site 2**		***11β-HSD-2*** **score at site 3**		***11β-HSD-2*** **score at site 4**		***11β-HSD-2*** **score at site 5**	
	**Estimate (SE)**	**P-value**	**Estimate (SE)**	**P-value**	**Estimate (SE)**	**P-value**	**Estimate (SE)**	**P-value**	**Estimate (SE)**	**P-value**
**Waist circumference (cm)** (Total number of observations=415; of which 43 (18.78%) subjects had one measurement, and 186 (81.22%) subjects had two measurements)										
**Model 1**	-0.3822 (1.0424)	0.7142	-0.08657 (0.7980)	0.9137	0.2635 (0.9701)	0.7862	0.5264 (0.7690)	0.4943	0.2132 (0.7252)	0.7690
**Model 2**	-1.1584 (1.0033)	0.2494	0.2359 (0.7715)	0.7601	0.6570 (0.9358)	0.4833	0.5122 (0.7455)	0.4928	-0.1516 (0.6976)	0.8281
**Systolic blood pressure (mmHg)** (Total number of observations=415; of which 43(18.78%) subjects had one measurement, and 186 (81.22%) subjects had two measurements)										
**Model 1**	-1.6096 (0.8326)	0.0545	-0.7568 (0.6372)	0.2362	1.2770 (0.7754)	0.1010	0.3766 (0.6161)	0.5416	-0.4901 (0.5780)	0.3974
**Model 2**	-1.6853 (0.8414)	0.0464	-0.6070 (0.6463)	0.3487	1.6432 (0.7850)	0.0375	-0.2687 (0.6286)	0.6695	-0.4886 (0.5828)	0.4027
**Diastolic blood pressure (mmHg)** (Total number of observations=415; of which 43 (18.78%) subjects had one measurement, and 186 (81.22%) subjects had two measurements)										
**Model 1**	-0.9251 (0.5519)	0.0951	-0.8601 (0.4222)	0.0428	0.3540 (0.5143)	0.4920	0.4535 (0.4092)	0.2690	-0.01360 (0.3827)	0.9717
**Model 2**	-1.0194 (0.5506)	0.0654	-0.8295 (0.4225)	0.0509	0.6060 (0.5139)	0.2395	0.07628 (0.4130)	0.8537	-0.05509 (0.3803)	0.8850
**Log transformed fasting glucose(mg/dL)** (Total number of observations=412; of which 46 (20.09%) subjects had one measurement, and 183 (79.91%) subjects had two measurements)										
**Model 1**	-0.00076 (0.007513)	0.9193	0.001955 (0.005764)	0.7348	0.006329 (0.006998)	0.3668	-0.01869 (0.005586)	0.0010*	0.002692 (0.005216)	0.6064
**Model 2**	0.007693 (0.007835)	0.3273	-0.00143 (0.006025)	0.8121	0.004141 (0.007304)	0.5713	-0.02250 (0.005876)	0.0002*	0.006799 (0.005426)	0.2116
**Log transformed high-density lipoprotein cholesterol (mg/dL)** (Total number of observations=412; of which 46 (20.09%) subjects had one measurement, and 183 (79.91%) subjects had two measurements)										
**Model 1**	0.002550 (0.01874)	0.8919	-0.00550 (0.01438)	0.7026	-0.00829 (0.01745)	0.6351	-0.01132 (0.01390)	0.4161	0.005434 (0.01303)	0.6770
**Model 2**	0.02022 (0.02000)	0.3132	-0.01804 (0.01539)	0.2423	-0.01149 (0.01865)	0.5386	-0.02960 (0.01489)	0.0481	0.01555 (0.01390)	0.2645
**Log transformed triglycerides (mg/dL)** (Total number of observations=412; of which 46 (20.09%) subjects had one measurement, and 183 (79.91%) subjects had two measurements)										

**Model 1**	0.02425 (0.04126)	0.5572	0.03580 (0.03163)	0.2588	0.004623 (0.03838)	0.9042	0.01794 (0.03047)	0.5566	-0.00972 (0.02872)	0.7354
**Model 2**	0.01567 (0.04011)	0.6964	0.03023 (0.03086)	0.3282	0.008336 (0.03740)	0.8238	0.02187 (0.02995)	0.4660	-0.01640 (0.02784)	0.5564

- 11β-hydroxysteroid dehydrogenase type 2 (*11β-HSD-2*)
- Model 1 includes *11β-HSD-2* z-scores for CpG sites 1, 2, 3, 4, and 5 as fixed effects and compound symmetry matrix structure to model the covariance structure of the repeated measurements for each outcome
- Model 2 is additionally adjusted for the following fixed effects: age, sex, and pubertal onset

* $p < 0.008$

Supplementary Table S7: Associations between DNAm z-score at *H19* and Repeated Measures of Cardiometabolic Risk Factors using Mixed-effects Models (*n*=245):

	***H19* score at site 1**		***H19* score at site 2**		***H19* score at site 3**		***H19* score at site 4**	
	**Estimate (SE)**	**P-value**	**Estimate (SE)**	**P-value**	**Estimate (SE)**	**P-value**	**Estimate (SE)**	**P-value**
**Waist circumference (cm)** (Total number of observations=446; of which 44 (17.96%) subjects had one measurement, and 201 (82.04%) subjects had two measurements)								
**Model 1**	-2.0199 (2.3712)	0.3951	0.4201 (0.9290)	0.6515	-0.1023 (1.0516)	0.9226	1.7246 (2.3175)	0.4575
**Model 2**	-0.4958 (2.2859)	0.8285	0.07485 (0.9050)	0.9342	-0.01468 (1.0179)	0.9885	1.0578 (2.2350)	0.6364
**Systolic blood pressure (mmHg)** (Total number of observations=446; of which 44 (17.96%) subjects had one measurement, and 201 (82.04%) subjects had two measurements)								
**Model 1**	2.9254 (1.9347)	0.1318	0.5769 (0.7460)	0.4402	-0.3888 (0.8519)	0.6485	-2.0551 (1.8928)	0.2786
**Model 2**	2.1289 (2.0092)	0.2905	0.6308 (0.7785)	0.4188	-0.3848 (0.8852)	0.6643	-1.4683 (1.9667)	0.4561
**Diastolic blood pressure (mmHg)** (Total number of observations=446; of which 44 (17.96%) subjects had one measurement, and 201 (82.04%) subjects had two measurements)								
**Model 1**	1.9218 (1.3080)	0.1430	0.07059 (0.5004)	0.8880	-0.1770 (0.5740)	0.7580	-1.1655 (1.2803)	0.3635
**Model 2**	1.7656 (1.3295)	0.1855	0.07927 (0.5086)	0.8763	-0.1730 (0.5822)	0.7667	-1.0460 (1.3026)	0.4228
**Log transformed fasting glucose(mg/dL)** (Total number of observations=443; of which 47 (19.18%) subjects had one measurement, and 198 (80.82%) subjects had two measurements)								
**Model 1**	-0.00046 (0.01808)	0.9796	0.002770 (0.006771)	0.6829	0.004283 (0.007883)	0.5875	-0.00728 (0.01773)	0.6816
**Model 2**	-0.01426 (0.01900)	0.4536	0.006626 (0.007262)	0.3627	0.001784 (0.008333)	0.8307	-0.00365 (0.01861)	0.8448
**Log transformed high-density lipoprotein cholesterol (mg/dL)** (Total number of observations=443; of which 47 (19.18%) subjects had one measurement, and 198 (80.82%) subjects had two measurements)								
**Model 1**	0.08297 (0.04325)	0.0562	-0.00522 (0.01640)	0.7507	-0.00854 (0.01895)	0.6527	-0.09188 (0.04237)	0.0311
**Model 2**	0.04381 (0.04758)	0.3582	0.009569 (0.01869)	0.6091	-0.01912 (0.02112)	0.3663	-0.08270 (0.04654)	0.0769
**Log transformed triglycerides (mg/dL)** (Total number of observations=443; of which 47 (19.18%) subjects had one measurement, and 198 (80.82%) subjects had two measurements)								
**Model 1**	-0.09145 (0.09240)	0.3233	0.03856 (0.03589)	0.2838	-0.02407 (0.04087)	0.5566	0.1266 (0.09037)	0.1626
**Model 2**	-0.04746 (0.08976)	0.5975	0.03792 (0.03481)	0.2772	-0.02790 (0.03962)	0.4820	0.09088 (0.08786)	0.3020

- Model 1 includes *H19* z-scores for CpG site 1, 2, 3, and 4 as fixed effects, and compound symmetry matrix structure to model the covariance structure of the repeated measurements for each outcome

- Model 2 is additionally adjusted for the following fixed effects: age and sex

* $p < 0.008$

Supplementary Table S8: Associations between DNAm z-score at *H19* and Repeated Measures of Cardiometabolic Risk Factors using Mixed-effects Models after Removing Outlier DNAm Value (*n*=244):

	***H19* score at site 1**		***H19* score at site 2**		***H19* score at site 3**		***H19* score at site 4**	
	**Estimate (SE)**	**P-value**	**Estimate (SE)**	**P-value**	**Estimate (SE)**	**P-value**	**Estimate (SE)**	**P-value**
**Waist circumference (cm)** (Total number of observations=444; of which 44 (18.03%) subjects had one measurement, and 200 (81.97%) subjects had two measurements)								
**Model 1**	-1.9711 (2.3738)	0.4072	1.7287 (1.9031)	0.3646	-0.9859 (1.5380)	0.5221	1.8050 (2.3215)	0.4376
**Model 2**	-0.4707 (2.2913)	0.8374	0.5902 (1.8478)	0.7497	-0.3633 (1.4905)	0.8076	1.0906 (2.2422)	0.6271
**Systolic blood pressure (mmHg)** (Total number of observations=444; of which 44 (18.03%) subjects had one measurement, and 200 (81.97%) subjects had two measurements)								
**Model 1**	2.9096 (1.9392)	0.1348	0.1319 (1.5394)	0.9318	-0.08892 (1.2463)	0.9432	-2.0820 (1.8985)	0.2739
**Model 2**	2.1309 2.0126	0.2909	0.5685 (1.6014)	0.7230	-0.3428 (1.2944)	0.7914	-1.4729 (1.9721)	0.4559
**Diastolic blood pressure (mmHg)** (Total number of observations=444; of which 44 (18.03%) subjects had one measurement, and 200 (81.97%) subjects had two measurements)								
**Model 1**	1.9019 (1.3105)	0.1480	-0.5299 (1.0351)	0.6092	0.2280 (0.8388)	0.7860	-1.2025 (1.2836)	0.3498
**Model 2**	1.7543 (1.3311)	0.1889	-0.4459 (1.0506)	0.6717	0.1809 (0.8503)	0.8317	-1.0820 (1.3054)	0.4081
**Log transformed fasting glucose(mg/dL)** (Total number of observations=441; of which 47 (19.26%) subjects had one measurement, and 197 (80.74%) subjects had two measurements)								
**Model 1**	-0.00042 (0.01812)	0.9813	0.004047 (0.01413)	0.7749	0.003419 (0.01151)	0.7667	-0.00720 (0.01778)	0.6859
**Model 2**	-0.01405 (0.01903)	0.4610	0.01456 (0.01502)	0.3333	-0.00357 (0.01217)	0.7696	-0.00310 (0.01866)	0.8684
**Log transformed high-density lipoprotein cholesterol (mg/dL)** (Total number of observations=441; of which 47 (19.26%) subjects had one measurement, and 197 (80.74%) subjects had two measurements)								
**Model 1**	0.08260 (0.04335)	0.0579	-0.01677 (0.03406)	0.6229	-0.00074 (0.02769)	0.9788	-0.09260 (0.04249)	0.0303
**Model 2**	0.04405 (0.04770)	0.3568	0.01789 (0.03828)	0.6407	-0.02474 (0.03092)	0.4245	-0.08213 (0.04670)	0.0799
**Log transformed triglycerides (mg/dL)** (Total number of observations=441; of which 47 (19.26%) subjects had one measurement, and 197 (80.74%) subjects had two measurements)								
**Model 1**	-0.08894 (0.09237)	0.3365	0.1086 (0.07369)	0.1420	-0.07136 (0.05967)	0.2329	0.1309 (0.09040)	0.1488
**Model 2**	-0.04632 (0.08982)	0.6066	0.08574 (0.07154)	0.2319	-0.06016 (0.05788)	0.2997	0.09421 (0.08801)	0.2854

- Model 1 includes *H19* z-scores for CpG site 1, 2, 3, and 4 as fixed effects, and compound symmetry matrix structure to model the covariance structure of the repeated measurements for each outcome
- Model 2 is additionally adjusted for the following fixed effects: age and sex

* $p < 0.008$

Supplementary Table S9: Associations between DNAm z-score at *H19* and Repeated Measures of Cardiometabolic Risk Factors using Mixed-effects Models Adjusting for Pubertal Onset (*n*=245):

	***H19* score at site 1**		***H19* score at site 2**		***H19* score at site 3**		***H19* score at site 4**	
	**Estimate (SE)**	**P-value**	**Estimate (SE)**	**P-value**	**Estimate (SE)**	**P-value**	**Estimate (SE)**	**P-value**
**Waist circumference (cm)** (Total number of observations=446; of which 44 (17.96%) subjects had one measurement, and 201 (82.04%) subjects had two measurements)								
**Model 1**	-2.0199 (2.3712)	0.3951	0.4201 (0.9290)	0.6515	-0.1023 (1.0516)	0.9226	1.7246 (2.3175)	0.4575
**Model 2**	-0.4601 (2.2842)	0.8405	0.1017 (0.9050)	0.9106	0.02333 (1.0184)	0.9817	1.0305 (2.2331)	0.6449
**Systolic blood pressure (mmHg)** (Total number of observations=446; of which 44 (17.96%) subjects had one measurement, and 201 (82.04%) subjects had two measurements)								
**Model 1**	2.9254 (1.9347)	0.1318	0.5769 (0.7460)	0.4402	-0.3888 (0.8519)	0.6485	-2.0551 (1.8928)	0.2786
**Model 2**	2.2231 (1.9256)	0.2495	0.8875 (0.7469)	0.2360	-0.1066 (0.8494)	0.9003	-1.6144 (1.8851)	0.3926
**Diastolic blood pressure (mmHg)** (Total number of observations=446; of which 44 (17.96%) subjects had one measurement, and 201 (82.04%) subjects had two measurements)								
**Model 1**	1.9218 (1.3080)	0.1430	0.07059 (0.5004)	0.8880	-0.1770 (0.5740)	0.7580	-1.1655 (1.2803)	0.3635
**Model 2**	1.7623 (1.2916)	0.1737	0.2580 (0.4953)	0.6030	-0.01024 (0.5665)	0.9856	-1.1167 (1.2655)	0.3784
**Log transformed fasting glucose(mg/dL)** (Total number of observations=443; of which 47 (19.18%) subjects had one measurement, and 198 (80.82%) subjects had two measurements)								
**Model 1**	-0.00046 (0.01808)	0.9796	0.002770 (0.006771)	0.6829	0.004283 (0.007883)	0.5875	-0.00728 (0.01773)	0.6816
**Model 2**	-0.01454 (0.01894)	0.4434	0.008114 (0.007262)	0.2652	0.003016 (0.008321)	0.7174	-0.00410 (0.01855)	0.8254
**Log transformed high-density lipoprotein cholesterol (mg/dL)** (Total number of observations=443; of which 47 (19.18%) subjects had one measurement, and 198 (80.82%) subjects had two measurements)								
**Model 1**	0.08297 (0.04325)	0.0562	-0.00522 (0.01640)	0.7507	-0.00854 (0.01895)	0.6527	-0.09188 (0.04237)	0.0311
**Model 2**	0.04758 (0.04618)	0.3039	0.01380 (0.01815)	0.4478	-0.01387 (0.02052)	0.4996	-0.08614 (0.04517)	0.0577
**Log transformed triglycerides (mg/dL)** (Total number of observations=443; of which 47 (19.18%) subjects had one measurement, and 198 (80.82%) subjects had two measurements)								
**Model 1**	-0.09145 (0.09240)	0.3233	0.03856 (0.03589)	0.2838	-0.02407 (0.04087)	0.5566	0.1266 (0.09037)	0.1626
**Model 2**	-0.04758 (0.08978)	0.5966	0.03750 (0.03488)	0.2834	-0.02833 (0.03968)	0.4760	0.09109 (0.08788)	0.3010

- Model 1 includes *H19* z-scores for CpG site 1, 2, 3, and 4 as fixed effects, and compound symmetry matrix structure to model the covariance structure of the repeated measurements for each outcome

- Model 2 is additionally adjusted for the following fixed effects: age, sex, and pubertal onset

* $p < 0.008$

Supplementary Table S10: Cross-sectional Associations between DNAm z-score at *PPAR- α* and Cardiometabolic Risk Factors using Linear Regression Adjusting for Pubertal Onset (*n*=345):

	***PPAR- α* score at site 1**		***PPAR- α* score at site 2**	
	**Estimate (SE)**	**P-value**	**Estimate (SE)**	**P-value**
**Waist circumference (cm)** (N= 345)				
**Model 1**	0.71915 (0.71474)	0.3150	-1.70941 (0.65445)	0.0094
**Model 2**	1.01378 (0.70496)	0.1513	-1.69631 (0.64590)	0.0090
**Systolic blood pressure (mmHg)** (N= 345)				
**Model 1**	0.58582 (0.60305)	0.3320	-1.02922 (0.55218)	0.0632
**Model 2**	0.50301 (0.58038)	0.3867	-0.67188 (0.53176)	0.2073
**Diastolic blood pressure (mmHg)** (N= 345)				
**Model 1**	0.58530 (0.42242)	0.1668	-0.57466 (0.38679)	0.1383
**Model 2**	0.59009 (0.40685)	0.1479	-0.34991 (0.37276)	0.3486
**Log transformed fasting glucose (mg/dL)** (N=310)				
**Model 1**	0.00598 (0.00614)	0.3305	0.00016627 (0.00600)	0.9779
**Model 2**	0.00293 (0.00609)	0.6309	0.00134 (0.00595)	0.8216
**Log transformed high-density lipoprotein cholesterol (mg/dL)** (N= 310				
**Model 1**	-0.00813 (0.01303)	0.5329	0.01206 (0.01273)	0.3445
**Model 2**	-0.00404 (0.01310)	0.7577	0.00824 (0.01281)	0.5205
**Log transformed triglycerides (mg/dL)** (N= 310)				
**Model 1**	0.01232 (0.03058)	0.6873	0.00118 (0.02989)	0.9684
**Model 2**	0.02099 (0.03062)	0.4936	-0.01145 (0.02995)	0.7026

- Peroxisome proliferator-activated receptor alpha (*PPAR-α*).
- Model 1 includes *PPAR- α* z-scores for CpG site 1 and 2
- Model 2 is additionally adjusted for age, sex, and pubertal onset

* $p < 0.008$

Supplementary Table S11: Primer Sequences and Details of CpG Sites Assessed [1]:

Gene or Element Name	# of CpG Sites Assessed	Loci of CpG Sites [a]	Primer Sequences			Locus of Amplified Region
			Forward	Reverse [b]	Sequencing	
LINE-1	4	Various [c]	TTGAGTTAGGTGTGG GATATAGTT	CAAAAAATCAAAAAAT TCCCTTTCC	AGGTGTGGAT ATAGT	Various[3]
11β-HSD-2	5	chr16: 67430541, 67430543, 67430562, 67430564, and 67430580	TTAAGTTTTGGAAGG AAAGGGAAAGA	ACATCCCCATACCCTTT ACTAATC	AGTTTTTGTTT TAGGTAGG	chr16: 67430512-67430745
H19	4	chr11: 2003031, 2003029, 2003027, and 2003024	TTTGTTGATTTTATTA AGGGAG	CTATAAATAAACCCCA ACCAAAC	GTGTGGAATT AGAAGT	chr11: 2002966-2003111
PPAR-α	2	chr22: 46149160 and 46149179	GGAGGTTTTTATGAG GATGTAGTT	ACACATATTAACCAAC AATAACTATCAT	GGATGTGGTT GTTTG	chr22: 46149046-46149244

Notes:

a. Loci are based off genome build GRCh38/hg38

b. All reverse primers for pyrosequencing are 5'biotinylated.

c. A consensus sequence found in all LINE-1s (located throughout the genome) is amplified and sequenced here. The specific sequence is as follows: 5'-

CTCGTGGTGCGCCGTTTCTTAAGCCG

Long interspersed nuclear elements (LINE-1); 11β-hydroxysteroid dehydrogenase type 2 (*11β-HSD-2*); Peroxisome proliferator-activated receptor alpha (*PPAR-α*).

Supplementary Table S12: Average DNAm z-score at LINE-1 [1] and Confounders Selection:

	Average DNAm z-score at LINE-1				P-value
	Q 1<br>*n*= 59	Q 2<br>*n*=61	Q 3<br>*n* = 61	Q 4<br>*n* = 61	
**Maternal Characteristics (at time of child's birth)**					
**Years of education,** %					
< 12 years	55.93	47.54	37.70	55.74	0.1872
12 years	25.42	40.98	45.90	34.43	
> 12 years	18.64	11.48	16.39	9.84	
**Age at childbirth,** (years)	26.83	27.07	25.93	27.39	0.5188
**Parity,** %					
1	33.90	31.15	47.54	36.07	0.1721
2	33.90	44.26	36.07	29.51	
≥ 3	32.20	24.59	16.39	34.43	
**Marital Status,** %					
Married	59.32	72.13	73.77	78.69	0.1149
Others (includes free union, single, separated, or divorced)	40.68	27.87	26.23	21.31	
**Enrollment in calcium supplementation study,** %					
Not enrolled	59.32	57.38	70.49	60.66	0.4494
Enrolled during pregnancy	40.68	42.62	29.51	39.34	
**Child Characteristics (at birth)**					
**Female, %**	70.00	52.46	52.46	39.34	0.0092*
**Gestation age,** (weeks)	38.81	38.82	38.97	38.75	0.9037
**Mode of delivery,** %					
Vaginal delivery	50.85	55.74	55.74	66.10	0.3939
C Section	49.15	44.26	44.26	33.90	
**Birth weight,** (kg)	3.15	3.13	3.20	3.13	0.8137
**Breastfeeding duration**, (weeks)	6.41	8.23	9.57	8.44	0.0171*
**Child Characteristics (at follow-up visits)**					
**Age,** (years)	9.65	10.32	10.73	10.63	0.0003*
**Metabolic equivalents,** (METs/week)	31.24	29.92	32.39	31.58	0.9341
**Pubertal onset, %**	25.00	44.26	57.38	42.62	0.0044*
**Total caloric intake,** (kcal/day)	2617.89	2778.31	2576.08	2578.95	0.3748

[1] Average DNAm at LINE-1 was computed as the average LINE-1 z-score at CpG site 1, 2, 3, and 4.
Means or percentages are presented for continuous or categorical variables, respectively.
Long interspersed nuclear elements (LINE-1)
* $p < 0.05$

Supplementary Table S13: Average DNAm z-score at *11β-HSD-2* [1] and Confounders Selection:

	**Average DNAm z-score at *11β-HSD-2***				**P-value**
	**Q 1** *n* = 61	**Q 2** *n* = 62	**Q 3** *n* = 62	**Q 4** *n* = 61	
**Maternal Characteristics (at time of child's birth)**					
**Years of education,** %					
< 12 years	55.74	48.39	46.77	45.90	0.3280
12 years	32.79	45.16	33.87	34.43	
> 12 years	11.48	6.45	17.74	19.67	
**Age at childbirth,** (years)	26.62	26.97	27.16	26.67	0.9460
**Parity,** %					
1	34.43	38.71	29.03	44.26	0.6075
2	40.98	32.26	41.94	29.51	
≥ 3	24.59	29.03	27.42	26.23	
**Marital Status,** %					
Married	72.13	64.52	72.58	75.41	0.5323
Others (includes free union, single, separated, or divorced)	27.87	35.48	25.81	24.59	
**Enrollment in calcium supplementation study,** %					
Not enrolled	57.38	66.13	62.90	60.66	0.6592
Enrolled during pregnancy	42.62	33.87	35.48	39.34	
**Child Characteristics (at birth)**					
**Female, %**	50.82	56.45	51.61	54.10	0.9223
**Gestation age,** (weeks)	38.83	39.13	38.52	38.90	0.1080
**Mode of delivery,** %					
Vaginal delivery	52.46	61.29	59.68	54.10	0.8987
C Section	45.90	38.71	38.71	44.26	
**Birth weight,** (kg)	3.16	3.15	3.11	3.18	0.8933
**Breastfeeding duration**, (weeks)	8.13	7.40	8.46	8.61	0.7751
**Child Characteristics (at follow-up visits)**					
**Age,** (years)	9.90	10.64	10.50	10.30	0.0466*
**Metabolic equivalents,** (METs/week)	29.31	29.89	34.25	32.04	0.5216
**Pubertal onset, %**	36.07	48.39	45.16	37.70	0.4510
**Total caloric intake,** (kcal/day)	2811.31	2640.92	2514.23	2580.73	0.1279

[1] Average DNAm at *11β-HSD-2* was computed as the average *11β-HSD-2* z-score at CpG site 1, 2, 3, 4 and 5.
Means or percentages are presented for continuous or categorical variables, respectively.
11β-hydroxysteroid dehydrogenase type 2 (*11β-HSD-2*)
* $p < 0.05$

Supplementary Table S14: Average DNAm z-score at *H19* [1] and Confounders Selection:

	Average DNAm z-score at *H19*				P-value
	**Q 1**<br>*n* = 61	**Q 2**<br>*n* = 61	**Q 3**<br>*n* = 62	**Q 4**<br>*n* = 61	
**Maternal Characteristics (at time of child's birth)**					
**Years of education,** %					
< 12 years	47.54	44.26	51.61	52.46	0.8105
12 years	37.70	42.62	35.48	31.15	
> 12 years	14.75	13.11	11.29	16.39	
**Age at childbirth,** (years)	26.48	26.69	26.98	27.07	0.9340
**Parity,** %					
1	49.18	31.15	32.26	34.43	0.0663
2	27.87	42.62	27.42	45.90	
≥ 3	22.95	26.23	38.71	19.67	
**Marital Status,** %					
Married	75.41	80.33	69.35	59.02	0.1071
Others (includes free union, single, separated, or divorced)	24.59	19.67	29.03	40.98	
**Enrollment in calcium supplementation study,** %					
Not enrolled	63.93	65.57	58.06	60.66	0.7226
Enrolled during pregnancy	36.07	34.43	40.32	39.34	
**Child Characteristics (at birth)**					
**Female, %**	49.18	52.46	50.00	60.66	0.5675
**Gestation age,** (weeks)	38.82	38.71	38.89	38.95	0.6168
**Mode of delivery,** %					
Vaginal delivery	62.30	59.02	58.06	47.54	0.6757
C Section	36.07	40.98	40.32	50.82	
**Birth weight,** (kg)	3.20	3.13	3.10	3.18	0.5830
**Breastfeeding duration**, (weeks)	9.43	8.38	6.89	8.03	0.0799
**Child Characteristics (at follow-up visits)**					
**Age,** (years)	10.59	10.65	10.28	9.86	0.0140*
**Metabolic equivalents,** (METs/week)	34.89	28.64	32.34	29.74	0.2589
**Pubertal onset, %**	47.54	40.98	48.39	31.15	0.1886
**Total caloric intake,** (kcal/day)	2581.31	2483.39	2709.46	2770.89	0.2434

[1] Average DNAm at *H19* was computed as the average *H19* z-score at CpG site 1, 2, 3, and 4.
Means or percentages are presented for continuous or categorical variables, respectively
* $p < 0.05$

Supplementary Table S15: Average DNAm z-score at *PPAR- α* [1] and Confounders Selection:

	Average DNAm z-score at *PPAR-α*				
	**Q 1**<br>*n* = 89	**Q 2**<br>*n* = 89	**Q 3**<br>*n* = 91	**Q 4**<br>*n* = 89	**P-value**
**Maternal Characteristics (at time of child's birth)**					
**Years of education,** %					
< 12 years	43.82	58.43	49.45	53.93	0.0766
12 years	40.45	29.21	42.86	26.97	
> 12 years	15.73	12.36	6.59	19.10	
**Age at childbirth,** (years)	26.38	27.04	26.36	25.93	0.5899
**Parity,** %					
1	44.94	37.08	40.66	32.58	0.1289
2	34.83	28.09	35.16	46.07	
≥ 3	20.22	34.83	23.08	21.35	
**Marital Status,** %					
Married	76.40	78.65	70.33	65.17	0.2510
Others (includes free union, single, separated, or divorced)	23.60	21.35	28.57	34.83	
**Enrollment in calcium supplementation study,** %					
Not enrolled	76.40	67.42	65.93	64.04	0.3655
Enrolled during pregnancy	23.60	32.58	32.97	35.96	
**Child Characteristics (at birth)**					
**Female, %**	51.69	44.94	52.75	57.30	0.4259
**Gestation age,** (weeks)	38.78	38.70	39.03	38.62	0.3877
**Mode of delivery,** %					
Vaginal delivery	58.43	49.44	61.54	65.17	0.3060
C Section	40.45	50.56	37.36	34.83	
**Birth weight,** (kg)	3.09	3.16	3.18	3.18	0.6758
**Breastfeeding duration**, (weeks)	8.20	9.12	7.41	7.75	0.1482
**Child Characteristics (at follow-up visits)**					
**Age,** (years)	14.49	14.39	13.84	13.73	0.0318*
**Metabolic equivalents,** (METs/week)	60.98	65.32	54.85	61.38	0.1592
**Pubertal onset, %**	92.13	94.38	92.31	92.13	0.9824
**Total caloric intake,** (kcal/day)	2423.71	2562.43	2144.12	2315.97	0.1417

[1] Average DNAm at *PPAR- α* was computed as the average *PPAR- α* z-score at CpG site 1, and 2.
Means or percentages are presented for continuous or categorical variables, respectively.
Peroxisome proliferator-activated receptor alpha (*PPAR-α*)
* $p < 0.05$

References

1. Wu, Y.; Goodrich, J.M.; Dolinoy, D.C.; Sanchez, B.N.; Ruiz-Narvaez, E.A.; Banker, M.; Cantoral, A.; Mercado-Garcia, A.; Tellez-Rojo, M.M.; Peterson, K.E. Accelerometer-measured Physical Activity, Reproductive Hormones, and DNA Methylation. *Med. Sci. Sports Exerc.* **2020**, *52*, 598-607, doi:10.1249/MSS.0000000000002175.
